# Building Surgical Research Capacity in Africa: The Ptolemy Project

**DOI:** 10.1371/journal.pmed.0030305

**Published:** 2006-07-11

**Authors:** Miliard Derbew, Massey Beveridge, Andrew Howard, Niall Byrne

## Abstract

The authors describe the Ptolemy Project, a recently developed model of electronic access to medical literature for surgeons in developing countries.

The Ptolemy Project ( www.ptolemy.ca) is a recently developed model of electronic access to medical literature for surgeons in developing countries. The program provides for East African surgeons to become research affiliates of the University of Toronto and have access to the full text resources of the university library, via a secure system that monitors and evaluates their usage [ 1].

The project started in 2001, and the beneficiaries of the project so far are 201 doctors, mostly belonging to the Association of Surgeons of East Africa ( Figure 1) and the College of Surgeons of East, Central, and Southern Africa (COSECSA). In this article, we describe an international collaboration program between the University of Toronto and East African surgical communities through the Ptolemy Project.

**Figure 1:**
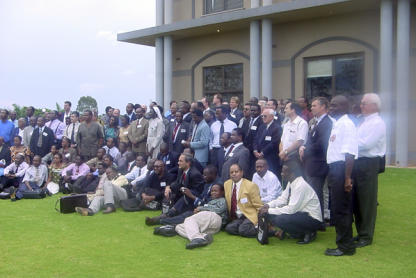
The 2005 Association of Surgeons of East Africa Meeting in Harare

## Bridging Knowledge and Research Gaps in East African Surgical Practice

Research is unthinkable without access to scientific literature. The Ptolemy Project attempts to bridge the gap between the literature available to researchers in wealthy developed countries and that available to researchers in developing countries, where researchers are largely deprived of access to essential health information. The project's aim is to improve access for developing country researchers to peer-reviewed medical literature, as well as to journals and documents originating in their part of the world. The Ptolemy Project is an electronic health information access tool designed to be effective, to be easy to use, and to satisfy the need for health information of surgeons in the developing world (K. Burton, A. Howard, M. Beveridge, unpublished data).

Health has improved in developing countries more rapidly over the last half century than it did in Western countries from the 17th century onwards [ 2]. The chief reason has been advances in technology and not, as many believe, improvement in income or education [ 2]. Technological advances take many forms: examples include vaccines against childhood diseases, effective short-term treatment of tuberculosis, and better information on the risks and control of diseases such as HIV/ AIDS and tobacco-related illnesses. None of the advances would have been possible without high-quality scientific research. Yet despite the promise of knowledge to reduce premature mortality and poverty, only 10% of the world's health research resources go towards the 90% of diseases that burden the poorest countries [ 3].

Making any serious improvement in mortality, morbidity, and disability among the global poor will require more locally driven collaborative research and wider usage of the scientific literature [ 4]. The fundamental idea underlying Ptolemy is that it is African doctors and researchers who know the right questions to ask in order to discover practicable solutions to the health problems of their regions. While Ptolemy alone is not sufficient to build a surgical research community, the electronic health information it provides is a fundamental and necessary component to this transformation.

Research capacity is lacking in the developing world, particularly in East Africa, making it vital that up-to-date research information is available to practicing physicians as a means to stimulate locally based and collaborative research. The need for the application of research information and the stimulation of research programs in East Africa is exemplified by the fact that a total of 400 surgeons are responsible for providing care to more than 200 million people. Isolation, burden of practice, and lack of research training and funding are the most common reasons for the dearth of research, and access by surgeons to contemporary scientific literature can help [ 5].

All of these problems bring dissatisfaction to doctors, and also to patients, who often travel days to reach medical care facilities only to be placed on a wait list. For example, in Ethiopia the waiting list for elective paediatric surgery/neurosurgery is as long as 8–10 months, and for other elective general surgery disciplines the waiting list is 6–8 months, irrespective of the disease pathology. The situation is similar in the other East African countries. Lack of resources makes surgical practice, surgical education, and research difficult in Africa [ 1].

## How Does Ptolemy Work?

Electronic media were introduced to East African surgeons in 2001. The Office of International Surgery at the University of Toronto has provided hands-on training on using these media to East African surgeons, as well as to the current trainees studying to take exams to become fellows or members of COSECSA. COSECSA candidates and surgical trainees are a prime target audience for Ptolemy, we believe, because those who learn to read the literature at an early stage in their careers are more likely to play leading roles in promoting education, research, and training in their regions. Surgeons in East Africa who want to sign up for Ptolemy download the registration and consent forms from the Ptolemy Web site and submit these to the Ptolemy coordinator in Tanzania or the Office of International Surgery in Toronto. The criteria to become a Ptolemy participant are shown in Box 1.

The Ptolemy Project also offers a reading course called “Surgery in Africa”, which is designed as a pilot project to train leaders in surgical education from Africa. “Surgery in Africa” is a self-directed, online, journal-based course primarily directed at surgical trainees who are undertaking the COSECSA Fellowship. The course is also available to all surgeons in the East African region, and internationally, who are interested in international surgery. The course started with extensive bibliographies on a selection of controversial topics relevant to practice in Africa, as well as a discussion forum for the participants. With the “Surgery in Africa” reading course, we hope to place online medical information at the disposal of African surgeons. The course reading materials are available online on the Office of International Surgery Web site ( http://www.utoronto.ca/ois/SIA.htm), which also has instructions on how to sign up for the full course materials.

## How Is Success Being Measured?

Participants' subjective satisfaction has been measured by an ongoing series of surveys, the results of which have been published in the BMJ [ 6]. Objective data regarding number of active users, their access locations, and journals and papers downloaded are collected from the library servers on a monthly basis. Beginning with 50 library accounts in December 2001, the number of Ptolemy accounts increased to 150 in 2003, and 300 in July 2005 (K. Burton, A. Howard, M. Beveridge, unpublished data). Of 167 registered participants in ten East African countries, 30 used the service between January 2005 and September 2005. In the broader field of 24 developing countries during this period, 56 users downloaded a total of 4,469 full-text papers from 672 different journals. The mean number of accesses per user was 79.8 and 14 users downloaded more than 100 papers each. The mean number of days users accessed Ptolemy was 15.5.

The top 20 journals accessed composed 41.4% of all accesses, and 75% of all users used at least some of the top 20 journals. Popularity of access via Ptolemy bore no relation to the published impact factors of the journals. The list of journals people actually read was far broader than the list generated from a previous self-report user survey. Examining titles of popular downloads suggests that clinical care for acute surgical problems is the most popular reason for consulting Ptolemy, and education is the next most popular. Box 2 shows the most frequently accessed journals. Note that most East African surgical journals are not available online, but are widely available locally in print form. A formal evaluation of the first four years of the Ptolemy Project is being conducted.

## Obstacles and Challenges

Surgeons who use Ptolemy use it frequently and extensively and read from a broad range of journals, both to support clinical care and to prepare educational material. Despite this, only 30 of the 167 registered participants in ten East African countries used the Ptolemy service between January 2005 and September 2005. Potential barriers to use include limited Web access, complexity of the Web, unfamiliarity with the Web, poor user support, difficulty in finding relevant information, a preference for alternative information sources, and a lack of perceived need to access the literature. Those who do use Ptolemy, use it a lot, which suggests they do find it useful, but it appears that there remain significant barriers to uptake of what we continue to feel is a valuable resource.

We propose collaborative work through COSECSA to identify and address important barriers. Two measures have been taken to address the problems of Ptolemy. First, the Office of International Surgery now employs a full-time research assistant based in the office of COSECSA, in Arusha, Tanzania, who provides hands-on training on how to use Ptolemy to COSECSA fellows and in particular to COSECSA trainees. Second, the Office of International Surgery gives scholarships to African educators to train trainers in surgical education in their region. It remains to be seen how successfully these steps are in improving the use of Ptolemy in the target region.

## Conclusion

Research is unthinkable without access to scientific literature. East African surgeons work with very limited resources, both in terms of manpower and technology, and yet they face a huge burden of health problems. Electronic access to projects such as Ptolemy is likely to be of great help to East African surgeons involved in research, assisting them in dealing with the health problems of their region. Collecting data, scientifically analyzing it, and using it to tackle the regional health problems will contribute to global health improvement [ 6].

We hope in the near feature that several electronic resources similar to Ptolemy will emerge to serve the different regions of low-income countries. Ptolemy can be considered as a collaborative model for other countries or institutions in the developed world who wish to help the global poor.

**Box 1. Criteria for Participating in Ptolemy**

Participants must

1. Be surgeons working in countries ranked less than 65 on the annual World Health Organization Human Development Index (preference is given to surgeons in East Africa, particularly COSECSA Trainees and Fellows and members of the Association of Surgeons of East Africa; 167 of the 201 participants are from East Africa [Ethiopia, Kenya, Malawi, Mozambique, Tanzania, Uganda, Zambia, Zimbabwe, Madagascar, and Seychelles]).
2. Consent to electronic monitoring of their library usage.
3. Be prepared to enter into a research affiliation with the University of Toronto Office of International Surgery.
4. Have regular access to the Internet.
5. Agree to participate in surveys to assess their use of the service supplied.
6. Agree not to sell the information they obtain, redistribute it for financial gain, or allow others to use the service provided for financial gain.
7. Acknowledge that the University of Toronto Office of International Surgery retains the right to discontinue their access at any time without any form of compensation.

**Box 2. Most Frequently Accessed Journals**

1. *American Journal of Surgery* (401 papers accessed)
2. *Current Orthopedics* (359 papers)
3. *The British Journal of Surgery* (276 papers)
4. *The Journal of the American Association for Pediatrics* (265 papers)
5. *Burns* (257 papers)
6. *Surgical Endoscopy* (225 papers)

## Abbreviations

COSECSA: College of Surgeons of East
